# Circularly-polarized, semitransparent and double-sided holograms based on helical photonic structures

**DOI:** 10.1038/s41598-017-16517-9

**Published:** 2017-11-28

**Authors:** Junji Kobashi, Hiroyuki Yoshida, Masanori Ozaki

**Affiliations:** 1 Division of Electrical, Electronic and Information Engineering, Osaka University, 2-1 Yamadaoka Suita, Osaka, 565-0871 Japan; 2 PRESTO, Japan Science and Technology Agency (JST), 4-1-8 Honcho Kawaguchi, Saitama, 332-0012 Japan

## Abstract

Recent advances in nanofabrication techniques are opening new frontiers in holographic devices, with the capability to integrate various optical functions in a single device. However, while most efficient holograms are achieved in reflection-mode configurations, they are in general opaque because of the reflective substrate that must be used, and therefore, have limited applicability. Here, we present a semi-transparent, reflective computer-generated hologram that is circularly-polarization dependent, and reconstructs different wavefronts when viewed from different sides. The integrated functionality is realized using a single thin-film of liquid crystal with a self-organized helical structure that Bragg reflects circularly-polarized light over a certain band of wavelengths. Asymmetry depending on the viewing side is achieved by exploiting the limited penetration depth of light in the helical structure as well as the nature of liquid crystals to conform to different orientational patterns imprinted on the two substrates sandwiching the material. Also, because the operation wavelength is determined by the reflection band position, pseudo-color holograms can be made by simply stacking layers with different designs. The unique characteristics of this hologram may find applications in polarization-encoded security holograms and see-through holographic signage where different information need to be displayed depending on the viewing direction.

## Introduction

Holography is a field of optics, which through the design of transmitted or reflected amplitude and/or phase patterns, enables the reconstruction of arbitrary wavefronts^1,2^. Since its invention in 1948^1^, various types of holograms have been developed and commercialized for applications in imaging, measurement, and security^2–6^. While the first holograms were fabricated by physically recording interference fringes on photographic plates, computer-generated holograms (CGHs) are now widespread, where the hologram patterns are generated numerically and implemented independently in a material of choice. Recent advances in nanofabrication technologies are opening new frontiers, as ultra-compact devices with advanced, integrated optical functions (e.g., circular-polarization-dependent image generation) can be realized using the so-called meta-surface concept, in which engineered sub-wavelength structures are used to modulate the optical field^7–12^. Most CGHs proposed to date, however, are designed to operate for light illuminating the hologram from a single side. When the CGH is illuminated from the opposite side, a phase conjugate image is generated, because of the reversal in the light propagation direction. Furthermore, reflective CGHs, which are known to possess higher efficiencies in general, are often opaque due to the requirement of a reflective substrate, and therefore have limited applicability^10^.

In this study, we present a semi-transparent and reflective CGH that generates different wavefronts depending on the illumination side of the hologram. The device can be varied from fully reflecting to transmitting by the circular sense of the polarization illuminating the device, but only the reflected light is phase modulated, meaning that the device can be seen through under ambient conditions. To realize the device, we use a cholesteric liquid crystal (ChLC) in which the constituent rod-like molecules self-organize into a helical structure, and gives rise to a circular-polarization sensitive Bragg reflection band over wavelengths *n*_o_ *p*–*n*_e_ *p*, where *n*_o_ and *n*_e_ are the ordinary and extraordinary refractive indices, and *p* is the helical pitch^13^. We exploit the limited penetration depth of light in the helical structure as well as the fluid nature of LCs to conform to asymmetric boundary conditions to confer asymmetry in the reflection properties. Furthermore, we demonstrate chiral-binary, pseudo-color holograms by stacking several devices designed to operate at different wavelengths and for different circular polarizations. Because each layer is only several micrometers thick and can be polymerized into a film, the active layer thickness can be kept to less than 100 µm even in a stacked device, which is thin from a practical point of view. The unique characteristics of this hologram may find applications in polarization-encoded security holograms and holographic signage where different information need to be displayed depending on the viewing direction.

## Results

### Design and implementation of the circularly-polarized, double-sided hologram

**Figure 1:**
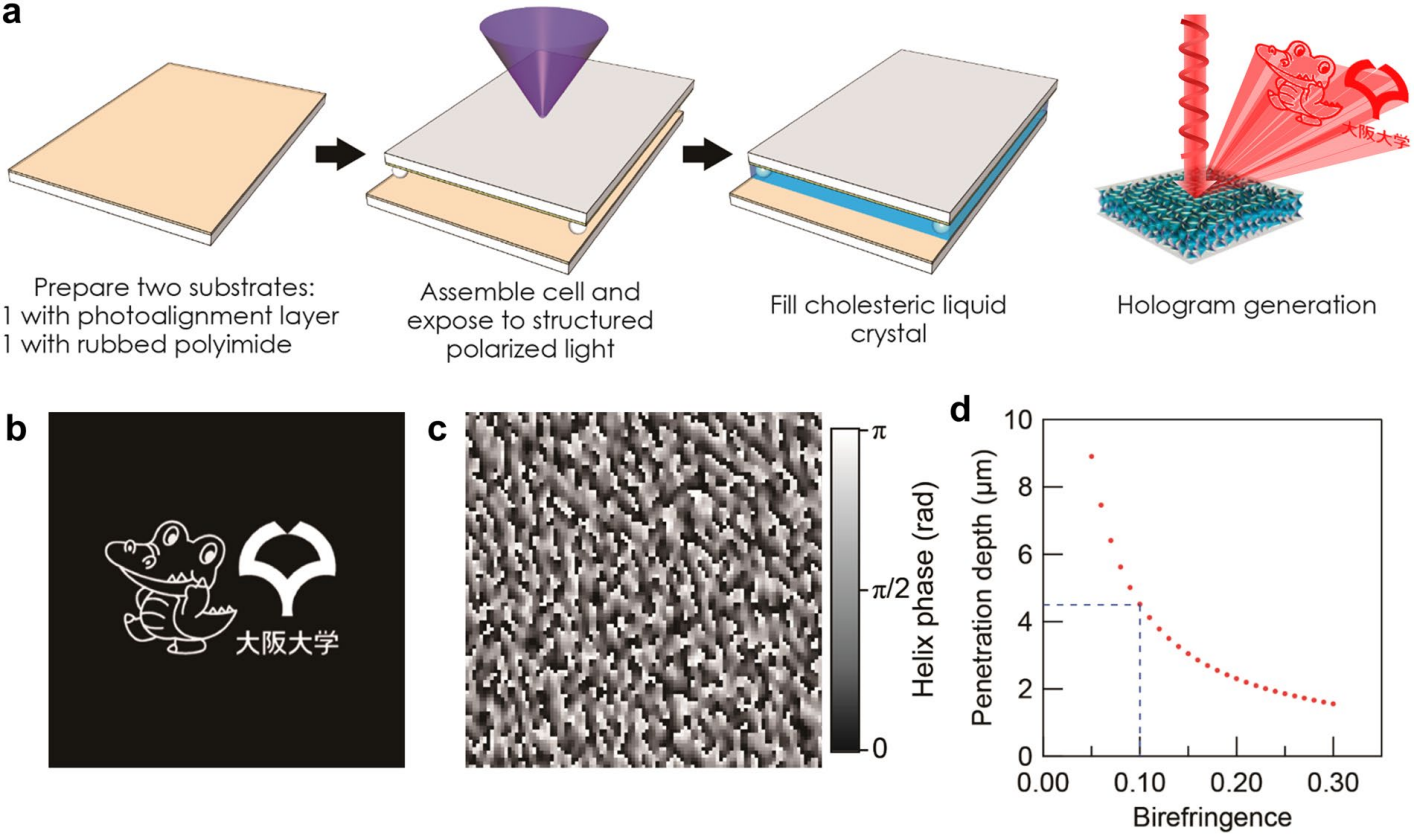
Cholesteric-liquid-crystal-based holograms. (**a**) Schematic image of the cholesteric-liquid-crystal-based hologram and its fabrication procedure. The device can also be fabricated by independently patterning two substrates coated with the photoalignment layer and assembling the cell after exposure. (**b**) Source image for hologram, with Osaka University mascot (Dr. Wani) and logo (used with permission from ©Osaka University). (**c**) A section of the helix phase profile (100 × 100 px of the 512 × 384 px-sized hologram) corresponding to the source image. (See Supplementary Supplementary Fig. S1 for the full phase profile). (**d**) Penetration depth of light along the helix axis, which is defined here as the length at which the intensity falls to 1%.

It has recently been shown that the phase of light reflected from a ChLC can be controlled by varying the geometric phase of the helical structure^14–17^. The reflected light phase varies by twice the helix phase, which means that a 0–π change in helix phase changes the light phase by 0–2π. The phenomenon is also polychromatic, occurring at all wavelengths falling within the Bragg reflection band^18,19^. With full-range control of the optical phase available, a CGH can be implemented in a ChLC device with planar alignment (i.e., a device with the helix axis along the substrate normal) by appropriately designing the helix phase distribution (Fig. 1a, most right). Figure 1b,c shows the image of “Dr. Wani” (Osaka University mascot) and the Osaka University logo, and the required helix phase profile calculated using the classical Gerchberg-Saxton (G-S) algorithm (see Supplementary Fig. S1 for the full design and generation methods)^20,21^. The linear relationship between the helix phase and optical phase enables retrieval of the required helix phase distribution by simply multiplying a factor of ±0.5 (where the sign depends on the handedness of the ChLC) to the optical phase profile obtained by the G-S algorithm.

The idea of creating a double-sided hologram stems from the fact that light with wavelength in the Bragg reflection band decays exponentially inside the ChLC^13^. Figure 1d shows the ChLC layer thickness at which the intensity of light at the center wavelength of the reflection band falls to 1% of the incident value, using values *n*_o_ = 1.5, *n*_e_ = *n*_o_ + Δ*n*, and *p* = 400 nm, where Δ*n* is the birefringence. Since conventional LC materials possess birefringence values of 0.1 and above, a ChLC layer of 4.5 µm can reflect more than 99% of the incident light. If the ChLC thickness is doubled, different patterns may be created on the two sides of the layer to reconstruct different wavefronts depending on the illumination side.

In practice, the ChLC will be sandwiched between two substrates with independent distributions of the orientational easy axis (Fig. 1a). Because ChLCs are viscoelastic, they can conform to asymmetric boundary conditions imposed in a slab type device by relaxing the helical structure. However, ChLCs under extremely strong confinement, such that the cell-gap is less than a single pitch have been predicted to show new order in which the uniaxial helical structure is no longer maintained^22^. Here, we confirm through hyperspectral imaging that for the samples fabricated here, where the patterning pitch was approximately six times the helical pitch, the helical axis remained in the cell-normal direction. Only small variations in pitch were observed, implying that the helix followed the anchoring on the top and bottom surfaces.

The proposed hologram was fabricated by imprinting the helix phase distribution shown in Fig. 1c on a glass substrate coated with a photoalignment layer through the use of a maskless photoalignment system^14,23,24^. The system was used to pattern the orientational easy axis at a pixel pitch of approximately 2.6 × 2.6 μm^2^. The counter substrate was coated with polyimide and rubbed unidirectionally, and the two substrates were assembled into a sandwich cell with a cell-gap of 9 µm. A left-handed ChLC material with a reflection band between 630 and 720 nm was infiltrated in the cell (its reflection spectrum is shown in Supplementary Fig. S2), and the cell was characterized by optical microscopy, spectroscopy, and laser illumination. Note that the pixel count of the hologram investigated by microscopy was set to 512 × 384 (1.35 × 1.01 mm^2^) to enable observation of the whole pattern by a low-magnification objective, while for laser measurements, a device with 1024 × 768 pixels was used to make the active area larger than the laser spot.

**Figure 2:**
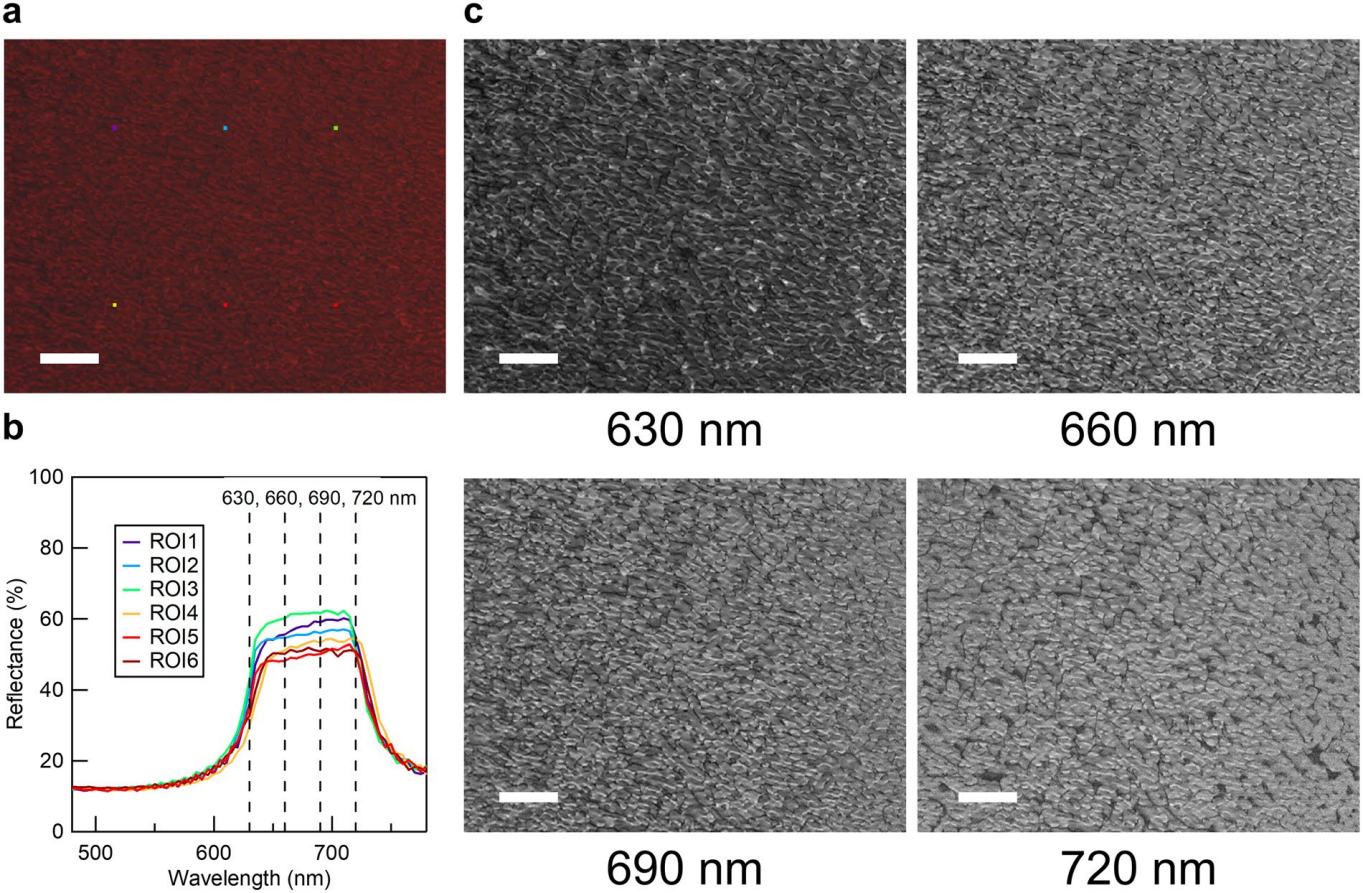
Experimental evaluation of pitch distribution in the hologram cell. (**a**) Combined color image of the sample generated from hyperspectral data. (**b**) Reflectance spectra averaged over an area of 6.6 × 6.6 μm^2^ at 6 different regions of interest (ROI) indicated by coloured squares in Fig. 2a. (**c**) Images at 4 different wavelength bands. Scale bar: 100 μm.

Figure 2a is an optical micrograph of the hologram, with artificial coloring generated from hyperspectral data. Figure 2b shows the reflectance spectra averaged over an area of 6.6 × 6.6 μm^2^ at six different regions of interest (ROI) in Fig. 2a, indicated by colored squares. At all ROIs, the reflection band has a similar profile to that of a homogeneous planar cell (Supplementary Fig. S2), but the center reflection wavelength varies between 680 ± 6 nm, implying that the helix axis is in the cell-normal direction, but is modulated in pitch between approximately 420 ± 4 nm. Images of the sample at four different wavelength bands (Fig. 2c, Δλ = 5 nm) confirm that regions with different helical pitch are distributed over the cell. The difference in reflectance depending on position is likely attributed to the difference in the Fresnel reflection depending on helix phase.

When the helix phase varies between ±π/2 against a unidirectionally-rubbed substrate, the number of helix turns varies between *d*/*p*_0_ ± 1/4, where *d* and *p*_0_ are the cell-gap and the natural pitch length. The pitch change also induces a slight change in the reflected phase, but for sufficiently large *d*, this effect is found to be insignificant. In our sample with *d* = 9 µm, the pitch variation was found to induce phase variations smaller than 0.06π, which is found to have a minute effect on the quality of the reconstructed images (Supplementary Fig. S3).

**Figure 3:**
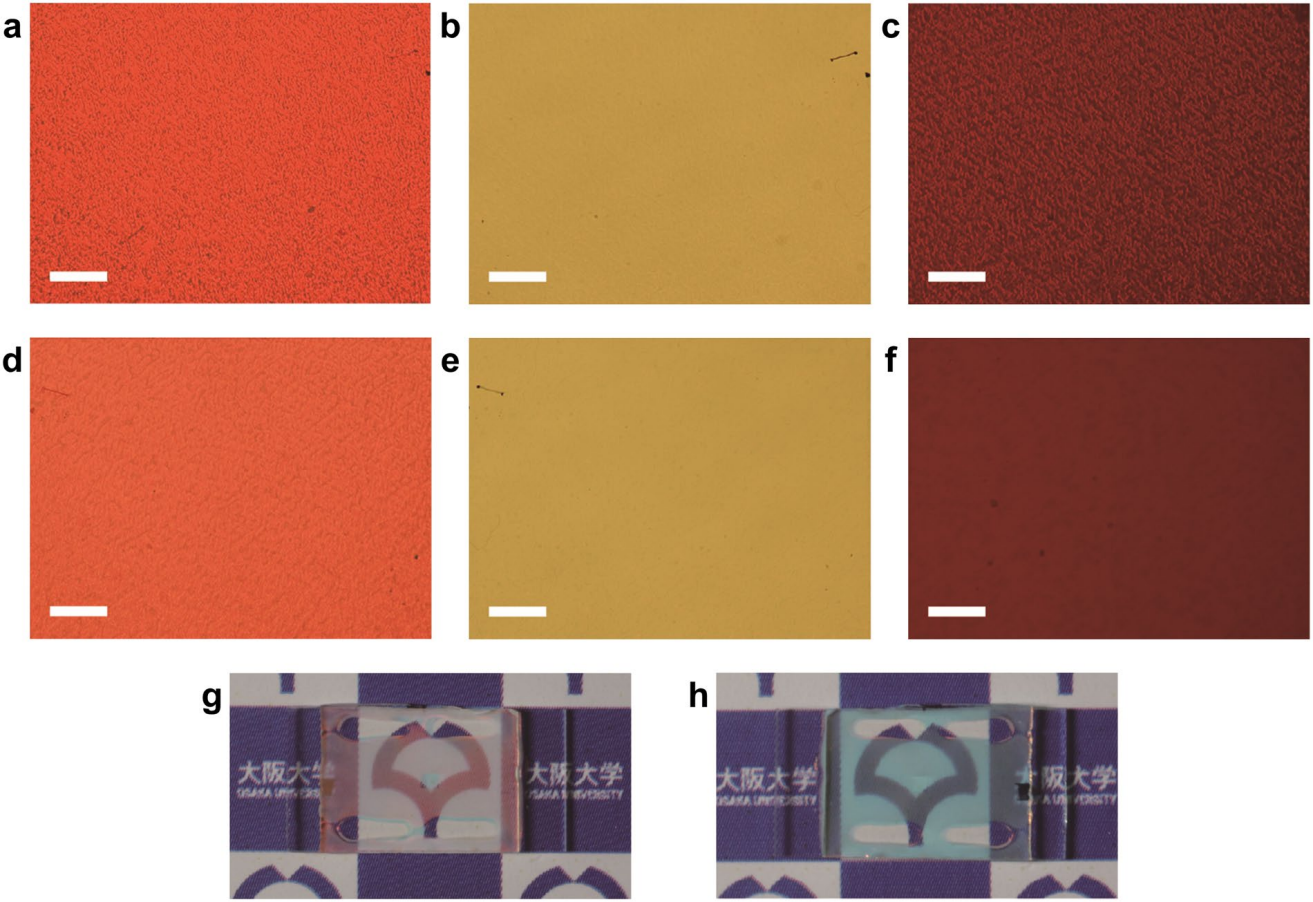
Optical textures of the double-sided hologram device. (**a**–**f**) Optical microscope images observed from the side with hologram (**a**–**c**) and uniform alignment (**d**–**f**). Scale bar: 200 μm. Images in reflection (**a**,**d**) and transmission (**b**,**e**), and reflective interferograms (**c**,**f**). (**g**,**h**) Appearance of the sample from the side with hologram (**g**) and uniform alignment (**h**).

Features similar to hyperspectral imaging are observed by standard optical microscopy, as shown in Fig. 3a–f. Figure 3a shows the sample observed in reflection mode from the side with the hologram pattern. Other than a variation in helical pitch, the patterning of the helix phase such that the orientation changes by multiples of π around a single point results in the appearance of orientational singular points^25^. However, such singularities were found to have negligible effects on the overall transmittance, as seen in the transmission-mode photographs of Fig. 3b and the transmittance spectra reported in Supplementary Fig. S4. Figure 3c is a microscopic interferogram of the sample, adjusted so that the whole field of view falls within one fringe. The hologram pattern becomes more pronounced, providing evidence that the reflected phase is indeed modulated by the helix phase. Figure 3d–f are microscopic images of the sample corresponding to observation modes of Fig. 3a–c, observed from the opposite side with uniform planar alignment. As suggested from numerical simulations, the reflected light only ‘sees’ the ChLC up to a certain penetration depth, and so the hologram pattern is almost invisible. Figure 3g,h show the appearance of the sample viewed from both sides. On the side with the hologram, the area outside the pattern scatters red light because the orientation is random, whereas the side with rubbing treatment shows a blue (cyan) texture, which is complementary to the red reflection, over the whole area. From both sides, the transparency of the sample is confirmed.

### Reconstruction of the chiral-encoded, double-sided hologram

**Figure 4:**
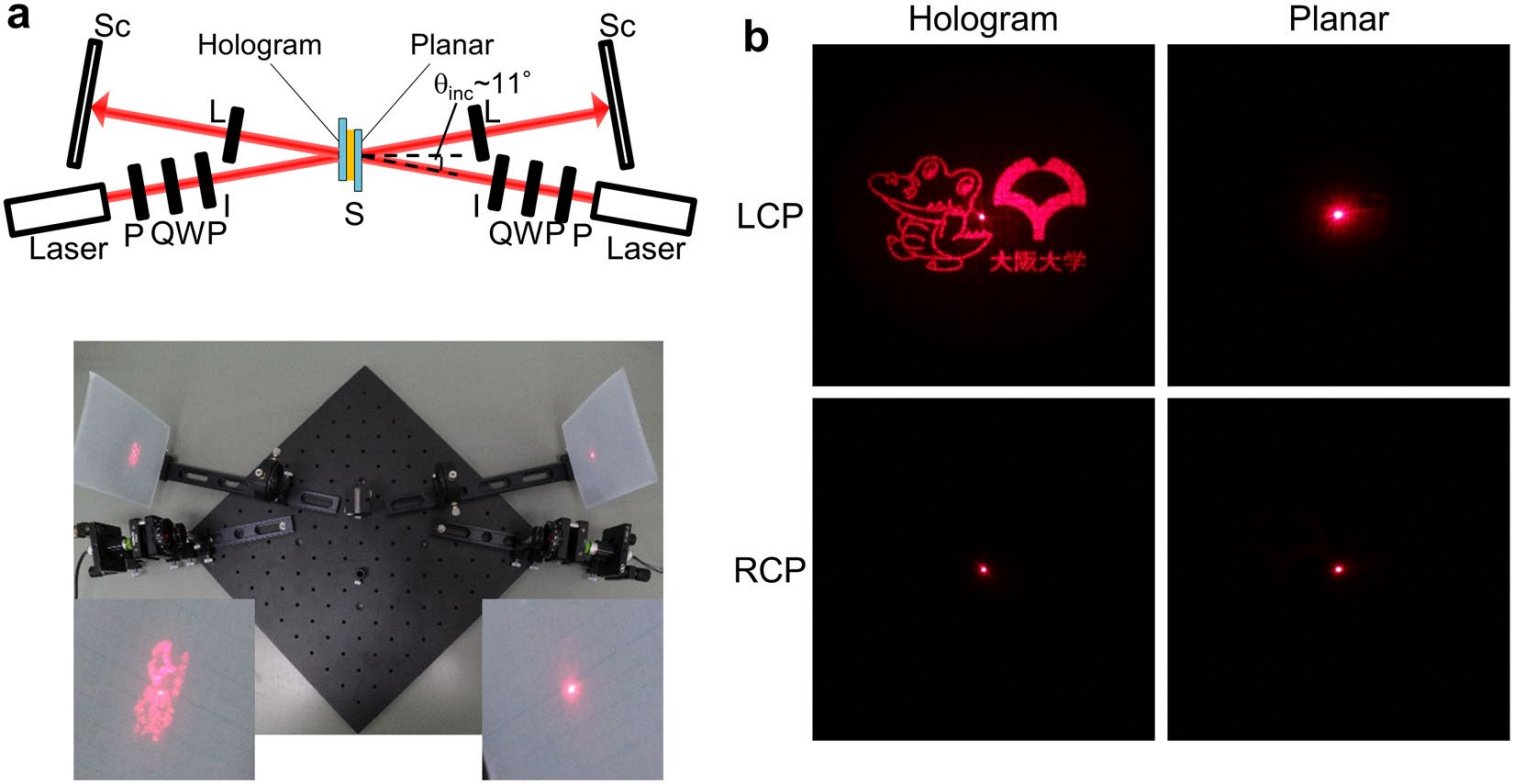
Reconstruction of the double-sided hologram. (**a**) Schematic image and picture of experimental setup. P: Polarizer; QWP: Quarter wave plate; I: Iris; S: Sample; L: Lens (*f* = 150 mm); Sc: Screen. The hologram image is observed only on the left screen, corresponding to the side with the hologram pattern. Inset: magnified view of screens. (**b**) Reflected light patterns on the screen upon illumination of the device from the hologram and planar sides, by left and right-handed circularly polarized light incidence (LCP/RCP).

A semiconductor laser (λ ~ 657 nm) was incident from the two sides of the film to evaluate the double-sided nature of the hologram. Figure 4a shows the experimental setup where the incidence angle of the lasers was set to approximately 11° to separate the transmitted beam from the reflected beam. The left side of the sample has the hologram pattern while the right side has uniform alignment, and the lasers are turned on either independently or simultaneously. Figure 4b is a photograph taken with the two lasers turned on; instantaneously one sees that the Osaka University mascot and logo appear only on the left screen. Figure 4c are close-ups of the holographic images projected on the screen, as the lasers are turned on independently, with different incident polarizations. At an incident angle of 11°, the ChLC still possesses strong circular polarization selectivity^18^, and shows a clear difference in intensity depending on the degree of circular polarization (left/right circular polarizations for maximum/minimum intensities). This results in a unique film-type device, in which a hologram is only visible from one side and can be switched on or off, depending on the illuminated polarization.

The conversion efficiency of the hologram was measured as the ratio of the light intensity in the hologram to the incident light intensity (for details of measurement, see Supplementary Fig. S5). The measured efficiency was 68% for LCP incidence, with major losses attributed to Fresnel reflection at the air/substrate interface, zero-order diffraction caused by the finite patterning pixel size and error in phase owing to the pitch distribution, and scattering due to local imperfections in the orientation. The relatively high conversion efficiency is due to the fact that the device is a pure phase-hologram with no absorption losses. As reported in Supplemnetary Figs S6 and S7, a comparable efficiency of 70% was obtained for the device with symmetric patterning, supporting that the limited penetration depth of light in the medium suppresses the deterioration in performance due to asymmetric patterning. It should be noted, however, that the efficiency will depend largely on the device parameters such as the LC refractive index and the thickness, and thinner cells are expected to give lower efficiencies in the double-sided device because a larger pitch variation would be induced, as discussed earlier. We believe further improvement will be possible through the improvement of patterning resolution, optimization of device structure such as through the use of anti-reflection coatings, and by pre-compensating the change in phase due to pitch variations in the hologram design.

### Fabrication of a pseudo-color, chiral binary, and double-sided hologram

**Figure 5:**
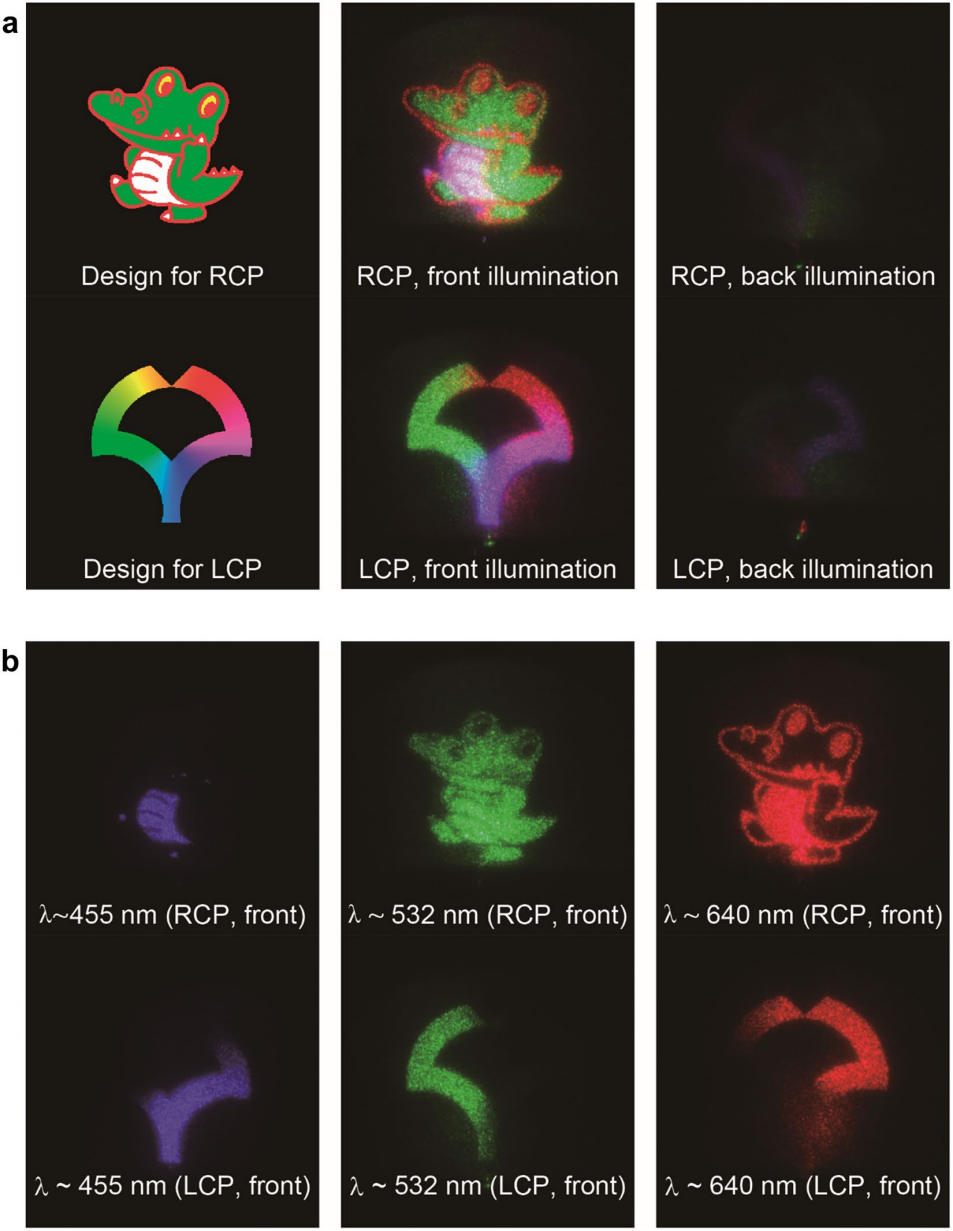
Pseudo-RGB color, double-sided hologram with chiral selectivity. (**a**) Design, and CP- and illumination-side-dependent far-field images generated from the hologram. Front and back illumination corresponds to illumination from the side with and without the hologram pattern. (**b**) Color resolved images generated from the hologram for front illumination.

The proposed device operates over a wavelength range that is determined by the refractive index and pitch of the LC. This range is typically several 10 nanometers and narrow compared to recently developed meta-surface holograms^9–12^; however, the strong color selectivity can be exploited to create pseudo-color holograms, by stacking several devices containing different patterns for different operation wavelengths. The principle can be further extended to a chiral binary device where both right- and left-handed ChLCs are stacked so that different wavefronts are generated depending on the circular sense of illuminated polarization^9,12^. Figure 5 demonstrates wavefront reconstruction from a pseudo-color, chiral binary, and double-sided hologram containing six ChLC layers, i.e., right- and left-handed ChLCs with pitch set to operate at red, green and blue wavelengths (for details of device design and fabrication, see Figs S8–S9). The top and bottom images of Fig. 5a correspond to images generated for right- and left-circular polarizations and, because all layers have asymmetric orientation patterns, the image is only generated from the hologram side (front illumination). Insertion of colour filters confirms that each layer is operating as designed (Fig. 5b), and the hologram image changes continuously when the circular sense of incident light is reversed by rotating a quarter wave plate (see movie S1). Being a stacked-film structure, the variation in diffraction angle depending on wavelength can be pre-compensated for each reflection band, providing an advantage for applications (Fig. S9).

## Discussion

The operating principle of our device makes it a computer-generated variant of the well-known Denisyuk or Lippmann hologram^3^. However, the simultaneous achievement of circular-polarization selectivity^9,12^ and asymmetry^4^ makes the proposed hologram distinct. Not only do these properties offer advanced functionality as security elements, but opens new possibilities for use as see-through holographic signage, where different information can be projected to the user, depending on the viewing side of the panel. Moreover, the fact that laser interference is not required for fabrication but self-organization of molecular materials is utilized opens the possibly of fabricating large-area color holograms by a roll-to-roll process. Future challenges remain to develop patterned alignment technology further to better control the orientation of the ChLC, in particular, the tilt angle with respect to the substrate. The hologram we created is a pure phase hologram, and a means to modulate the reflectance is necessarily for the reconstruction of the complex amplitude; this should be possible by using the recently discovered ‘heliconical’ ChLCs^26^, if the tilt angle in each pixel can be controlled to sustain a particular value. Exploring means of dynamic tuning is also important, either by tuning ChLCs directly by an electric field or through the use of field-sensitive alignment layers^27^. Finally, while we have exploited the self-organizing nature of ChLCs to fabricate the device, the same design principle can be applied to other helical photonic structures, such as chiral sculptured films^28^ and helix metamaterials^29^. By the appropriate choice of materials, similar devices should be realizable over a wide range of electromagnetic frequencies.

## Methods

### Calculation of the penetration depth

The penetration depth of light, defined here as the length of the ChLC medium required for the light intensity to fall to 1% of the incident intensity, was evaluated using a commercial finite element analysis software (COMSOL, Multiphysics). A 2-dimensional calculation was performed (in the *x*-*y* plane), where a right-handed ChLC with uniform helix phase and pitch of 400 nm was assumed to exist for 12 µm, with the helix axis in the *y*-direction. The ordinary refractive index of the ChLC was assumed to be *n*_o_ = 1.5, and the birefringence was varied from 0.05 to 0.30. Periodic boundary conditions were assumed in the x-direction and perfectly matched layer (PML) boundary conditions was set in the y-direction, and the electro-magnetic field distribution was calculated as a right-circularly polarized light was incident on the ChLC. The penetration depth was obtained by curve-fitting the square of electro-magnetic field profile with an exponential function.

### Materials

For the experiment in Figs 2–4, a left-handed ChLC material was prepared by mixing a host nematic liquid crystal (Merck, MLC-2140) and chiral dopant (HCCH, S-5011) at a weight ratio of 97.8:2.2. The reflection spectrum of the material, characterized in a 9-μm-thick sandwich cell with uniform planar alignment treatment is shown in Supplementary Fig. S2. The reflection band appeared between 634 and 724 nm. Using the Grandjean-Cano wedge method^30^, the pitch was found to be approximately 420 nm. A similar value is also obtained from optical calculations, substituting refractive indices of *n*_e_ = 1.7688 and *n*_o_ = 1.5158 (measured at 589.3 nm, 20 °C) from the datasheet of the host nematic LC into the equation giving the reflection band, *n*_o_ *p*–*n*_e_ *p*.

For fabrication of the pseudo-color hologram, precursors of a composite of photopolymerizable ChLC were prepared by mixing host nematic LC (Merck, MLC-2139), mesogen monomer (Merck, RM257), photoinitiator (BASF, Irgacure 819), left- (HCCH, S-5011) and right-handed (HCCH, R-5011) chiral dopants at the weight ratio of approximately 78:19:1:X; where X is 1.8–2.5 wt% to the host so that the reflection band existed at approximately 455, 532 and 640 nm, which were the main wavelengths of the laser used. The reflection spectrum of the materials after polymerization is shown in Supplementary Fig. S8. Three reflection bands appeared at approximate wavelength ranges of 450–470, 520–550, 620–660 nm.

### Fabrication of single-color holograms

The double-sided holograms were fabricated by preparing a substrate with the hologram pattern (“hologram substrate”) and a substrate with uniform alignment (“uniform substrate”), and assembling them into a sandwich cell with gap of 9 µm. The hologram substrate was prepared by coating a photoalignment agent (DIC, LIA-03), while the planar substrate was coated with a polyimide-based alignment agent (JSR, AL1254) and rubbed unidirectionally. After cell assembly, the orientational easy axis was patterned on the hologram substrate by irradiating linearly polarized light of various angles with an in-house built, maskless projection system^9^. The system comprises a LC display (LCD) projector and a rotating waveplate, and sequentially projects linearly polarized light at 3° increments (corresponding to 60 phase-levels) at positions predefined using a PC. The photoalignment layer is azobenzene-based, and sets the orientational easy axis parallel to the substrate, perpendicular to the incident polarization. The wavelength of the patterning light and typical dose for patterning were 436 nm and 18 mJ for each polarization angle. After patterning, the cell was injected with the ChLC material in the isotropic phase (100 °C) and cooled to room temperature, at which measurements were made.

The symmetric hologram was made in a similar manner to the double-sided hologram except that two hologram substrates were used instead of one.

### Evaluation of the helical pitch distribution

The helical pitch distribution was evaluated by acquiring hyperspectral data of the hologram cell. A multispectral camera (EBA Japan, NH7-YO) with a pixel resolution of 1280 × 1024 was equipped on a standard polarizing optical microscope (Nikon, Eclipse LV-100N POL) and measured in reflection mode, upon illuminating linearly polarized light. The distribution of the reflection band reported in the text (680 ± 6 nm) was evaluated as an average measured over an area of 6.6 × 6.6 μm^2^ (corresponding to 4 × 4 pixels) at 81920 positions in the hologram.

### Observation of texture and measurement of spectra

A polarizing optical microscope (Nikon, Eclipse LV-100 POL) was used to observe the optical texture of the samples. The reflectance and transmittance spectra were acquired with a spectrometer (PMA-11, Hamamatsu) coupled to the microscope by a bundled fiber with diameter of 1 mm and an objective lens with 10× magnification. The measurement spot was approximately 100 µm in diameter, which is larger than the patterning pitch (2.6 × 2.6 μm^2^). The interferogram of the cell was obtained with an in-house-built interferometric microscope^14^.

### Pseudo-color hologram fabrication and observation

Hologram substrates were fabricated for each ChLC precursor according to the design described in Supplementary Fig. S9. After assembly of the cell with a planar countersubstrate, the materials were injected into the cells and photopolymerized by irradiating UV light (365 nm, 220 mW/cm^2^) for 20 minutes. Afterwards, the cells were stacked to make a single, six-layered device. The stacking order, counting from the light source, was right-handed blue, green, red and left-handed blue, green, red ChLCs, where the color indicates the reflected wavelength.

For observation, three lasers for RGB color (center wavelengths: 455, 532, and 640 nm) were combined and irradiated at an incident angle of approximately 10°, and the reflected beam was projected on to a screen after passing through an imaging lens (*f* = 300 mm). The polarization state of the laser was controlled using a polarizer and a rotatable achromatic quarter wave plate.

## Electronic supplementary material

Supplementary information

Pseudo-color image generation from six-layered hologram device
