# Supplementary material for: Circularly-polarized, semitransparent and double-sided holograms based on helical photonic structures
Source: Sci Rep. 2017 Nov 28;7:16470. doi: 10.1038/s41598-017-16517-9 (PMC5705659; doi:10.1038/s41598-017-16517-9)
Supplement: Supplementary file 1 — Supplementary information [file 41598_2017_16517_MOESM1_ESM.pdf]

Supplementary Information for

Circularly-polarized, semitransparent, double-sided holograms based on helical photonic structures

Junji Kobashi,[1] Hiroyuki Yoshida, [1,2] and Masanori Ozaki[1]

[1] Division of Electrical, Electronic and Information Engineering, Osaka University, 2-1 Yamadaoka Suita, Osaka 565-0871, Japan.

[2] PRESTO, Japan Science and Technology Agency (JST), 4-1-8 Honcho Kawaguchi, Saitama 332-0012, Japan.

1. Design and generation of the hologram pattern

The hologram pattern was generated by the Gerchberg-Saxton (G-S) algorithm according to the following procedure [18]. First, the target image was prepared in Fourier space (Fig. S1a) and rescaled to fit the projection area, which has an aspect ratio of 4:3; the actual number of pixels used was either 512 × 384 px or 1024 × 768 px, depending on the desired size of the hologram (the size of a pixel realized experimentally is approximately 2.6 × 2.6 μm^2, yielding 1.35 × 1.01 mm^2 and 2.70 × 2.03 mm^2 as the hologram size). The source image was then inverse-Fourier-transformed to obtain a complex signal at the object plane, and its amplitude was replaced with a uniform signal before applying a Fourier transform to reproduce the image in the Fourier plane. The accuracy of image reproduction was improved by repetitively performing the procedure after replacing the amplitude of the image in the Fourier plane with the target image. The whole procedure was repeated until the zero mean normalized cross correlation (ZNCC) of the reproduced image to the original image was above 0.98. The final phase distribution and the reproduced image giving a ZNCC of 0.981 are shown in figs. S1b and S1c. In experiment in the manuscript, the phase distribution is converted to the helix phase distribution of ChLC by being multiplied by ±0.5 and rounded to have 60 levels with increment of 3°.

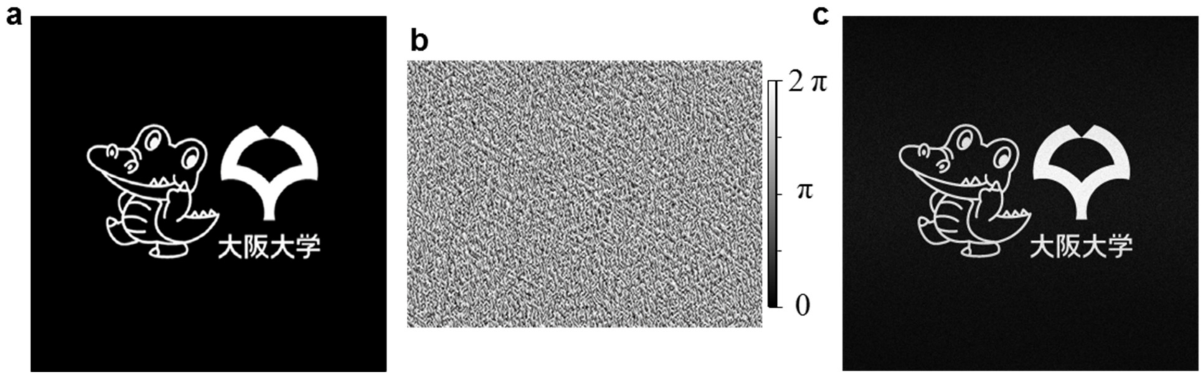

Fig. S1. Hologram design, calculated phase, and reconstructed image. a, Target image, **b**, Phase distribution, **c**, Simulated image.

2. Reflection spectrum of the ChLC material

The ChLC material used to make the hologram was characterized in a standard sandwich cell with uniform planar alignment treatment. Two substrates coated with photoalignment agent were assembled into a 9 μm-thick sandwich cell and irradiated upon linearly polarized UV light (365 nm, 100 mW/cm^2) to induce uniform planar alignment. The reflectance spectrum was acquired with a spectrometer (PMA-11, Hamamatsu) coupled to a polarizing optical microscope (Nikon, Eclipse LV-POL 100) by a bundled fibre with diameter of 1 mm and an objective lens with 10× magnification. As shown in Fig. S2, the reflection band appeared between 634 and 724 nm. Using the Grandjean-Cano wedge method, the pitch was found to be approximately 420 nm. A similar value is also obtained from optical calculations, substituting refractive indices n_e = 1.7688 and n_o = 1.5158 (measured at 589.3 nm, 20°C) from the datasheet of the host nematic LC into the equation for the reflection band, $n_o p - n_e p$.

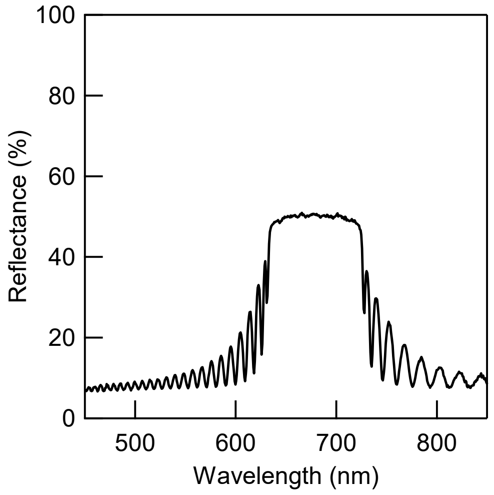

Fig. S2. Reflection spectrum of the ChLC material..

3. Effect of pitch distribution on the hologram quality

In the proposed hologram, the helix phase is varied through defining the orientational easy axis on a substrate. When the orientational easy axis of one substrate is varied with respect to the other while maintaining the cell-gap, the number of helix turns within the cell, and hence the helical pitch, changes. The helical pitch for a cell with an arbitrary orientational easy axis, directed at angle φ, with respect to the counter-substrate is given by the following equation:

$$p = \frac{d}{d / p_0 + \varphi / 2\pi}, \tag{1}$$

where d is the cell gap and p_0 is the pitch for parallel or antiparallel boundary condition (φ = 0 or π). Note that because of the head-tail symmetry, the pitch changes discontinuously at φ = ±π/2 so as to take a value that is close to the natural pitch, p, of the material, which is determined by the concentration of the chiral dopant. The largest pitch difference is obtained between regions with φ = π/2 and –π/2 and is given by:

$$\Delta p = \frac{d}{d / p_0 - 1/4} - \frac{d}{d / p_0 + 1/4} = \frac{d}{2} \frac{1}{(d / p_0)^2 - 1/16}. \tag{2}$$

From Eq. 2, one finds that the pitch difference decreases with an increase in the cell-gap. By substituting the experimental condition d = 9 μm and p_0 = 420 nm for this study, Δp is calculated to be 9.8 nm (p_0 ± 4.9 nm), agreeing satisfactorily with the experimental result in Fig. 2 in the manuscript.

The reflected light phase from a ChLC with varying helix pitch length and helix phase was calculated using Berreman's 4×4 matrix method [1]. In calculation a ChLC with the refractive indices n_e = 1.7688 and n_o = 1.5158 were sandwiched between two glass substrates (n = 1.53) separated by a gap of 9 μm. Fig. S3a shows a 2-dimensional image of the reflected phase, where the horizontal axis shows the helix pitch, the vertical axis shows the helix phase, and the colour shows the reflected light

phase. The white dashed line on the image indicates the pitch length of the sample when the boundary condition is varied from $\varphi = \pi/2$ to $-\pi/2$ in a cell with cell-gap of 9 μm. Fig. S3b plots the helix phase dependence of the reflected phase assuming a fixed pitch of 420 nm or variable pitch along the white dashed line in Fig. S3a. Although the two lines do not coincide, it is found that the pitch variation has a minute effect of $< 0.06\pi$ on the reflected light phase.

The effect of the pitch variation on the hologram quality can be quantitated by calculating the hologram pattern from the same helix phase distribution, but with modulated light phase (using the relationship between the reflected light phase and helix phase shown in Fig. S3b). Figs. S3c,d show simulated images for fixed pitch and variable pitch with 60 helix phase levels. Almost identical patterns are obtained, with ZNCCs of 0.980 and 0.978.

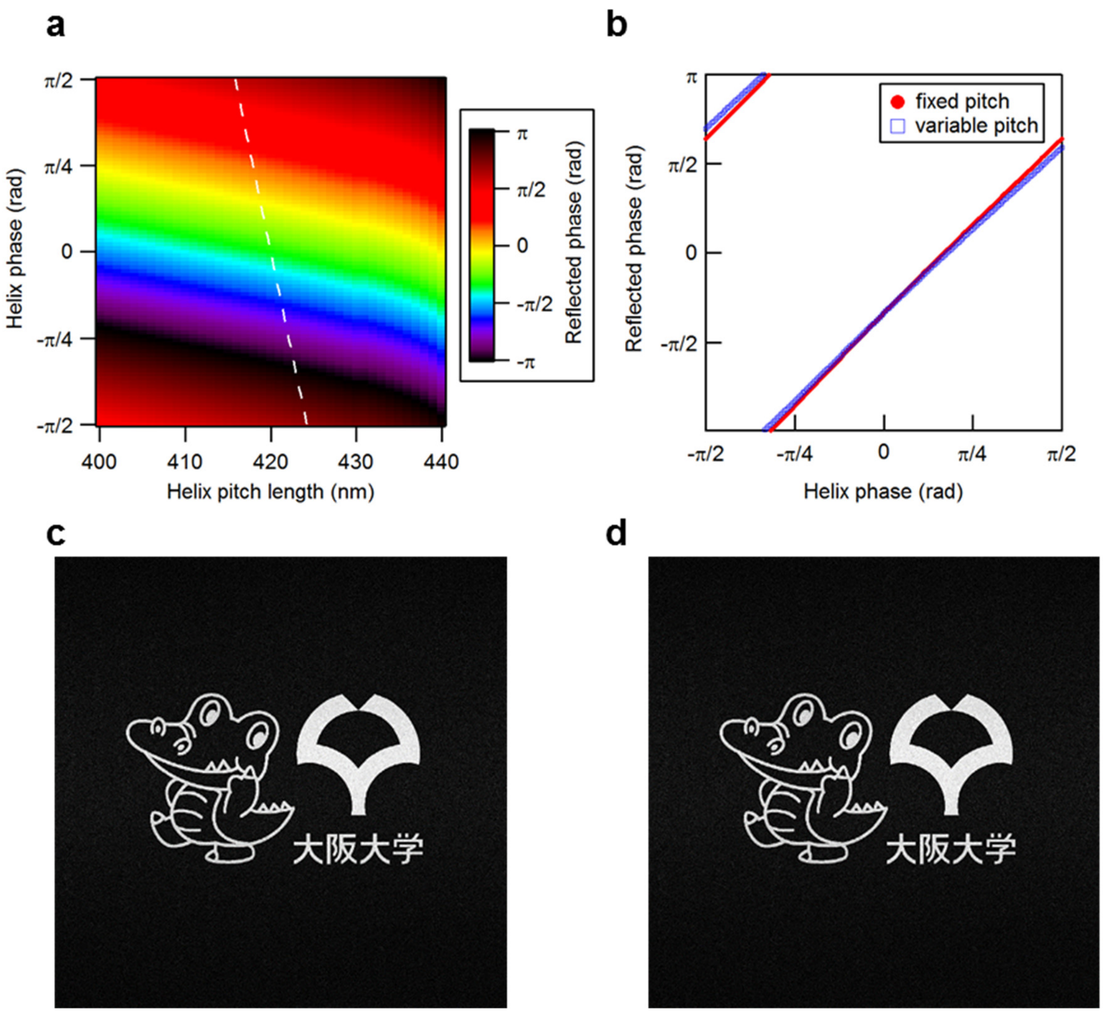

Fig. S3. Numerical simulations of the effect of pitch distribution on the hologram quality. a, Dependence of reflected phase on pitch length and phase of helix. **b**, The helix phase dependence of the reflected phase assuming a fixed pitch of 420 nm or variable pitch, **c,d,** Simulated hologram images with fixed (p_0=420 nm) (**c**) and variable pitch length (d).

4. Comparison of the transmittance spectrum of uniform planar and hologram ChLC devices

The transmittance of the ChLC cells were measured using the same setup as that for reflection measurements described in Section 2, but in transmission mode of the polarizing optical microscope. As shown in Fig. S4, the transmittance shows a decrease between approximately 630 and 720 nm, owing to the presence of the reflection band. The transmittance for both the planar and hologram samples are similar, indicating that the presence of the helix phase pattern has negligible effect on the transmittance of the sample. It is noted that the amplitude of the sidelobes (so-called Pendellösung oscillations) is smaller in the sample with the hologram pattern, as creating a phase distribution corresponds to making the reflection plane non-planar.

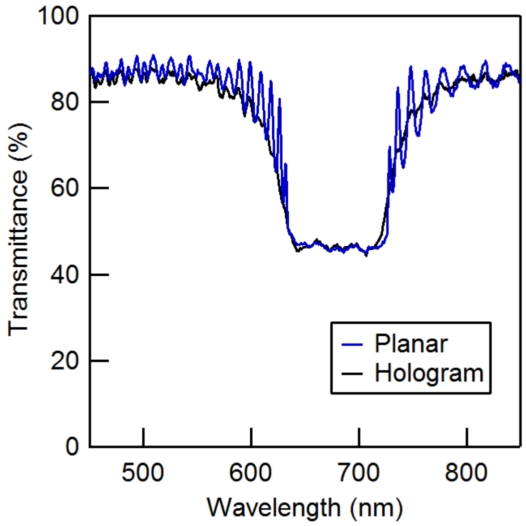

Fig. S4. Transmittance spectrum of ChLC devices with uniform planar and hologram alignment.

5. Experimental setup for the measurement of diffraction efficiency

Fig. S5 depicts the setup for measuring the conversion efficiency of the hologram. The setup is essentially the same as half of the setup used in Fig. 3 of the manuscript; an extra iris was placed after the cell to measure the intensity of the 0th order diffraction of hologram. The polarization state of the incident light was controlled to possess the same handedness as the ChLC helix using a polarizer and a quarter wave plate. The light intensity was measured at two points P1 and P2, and the conversion efficiency, η, was evaluated by the following equation:

$$\eta = \frac{I_2 - I_1}{I_0} \times 100[\%],$$

where I_0 is the incident light intensity at P1, I_1 is the 0th order intensity at P2, and I_2 is the total light intensity at P2.

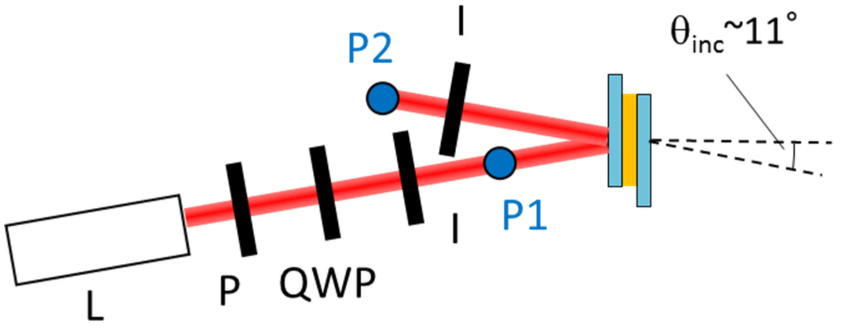

Fig. S5. Experimental evaluation of conversion efficiency. L: laser; P: Polarizer; QWP: Quarter wave plate; I: Iris; P1,2: Measurement points.

6. Comparison of double-sided and standard hologram devices

Here, the performance of the double-sided hologram device with asymmetric patterning (fabricated from a substrate with hologram pattern and the counter-substrate with uniform planar alignment) was compared with a standard device with symmetric patterning (same hologram pattern on both substrates). The reflectance and transmittance spectra of the devices were first compared from microscopic spectroscopy, using the same setup as that described in Section 2. The reflectance and transmittance spectra shown in figs. S6 a,b show that the asymmetry in the pattern has a negligible effect on the optical quality of the sample.

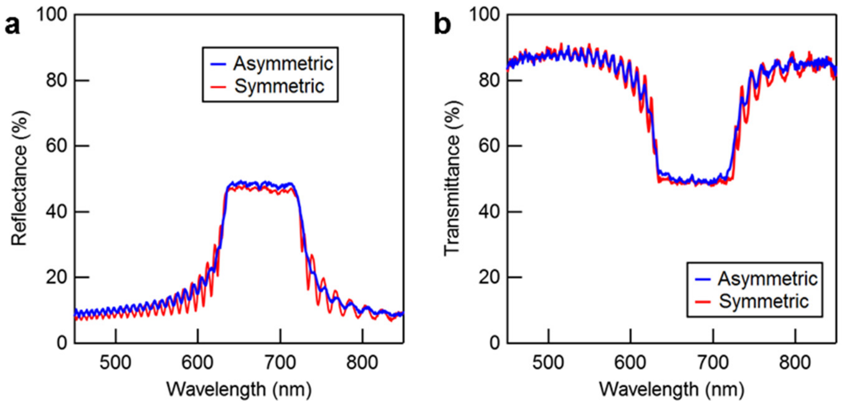

Fig. S6. Comparison of optical properties of hologram devices with asymmetric and symmetric patterning. a, Reflectance. **b**, Transmittance.

Figs. S7 a,b shows photos of illuminating the double-sided and standard hologram devices, using the same setup described in Fig. 3 of the manuscript. Unlike the double-sided device in which the image appears only from the side with the hologram pattern, the standard device generates an image from either side of the device. However, the image generated from the standard device becomes inverted on one side because the phase reverses sign as a result of creating the same pattern on both substrates [14]. Being able to suppress the appearance of such a conjugated image is an additional advantage of creating an asymmetric phase pattern. Moreover, the quality of the image from the two devices are

indistinguishable (compare Figs. S7 d,f), which again proves that effect of the pitch distribution on the hologram quality is insignificant.

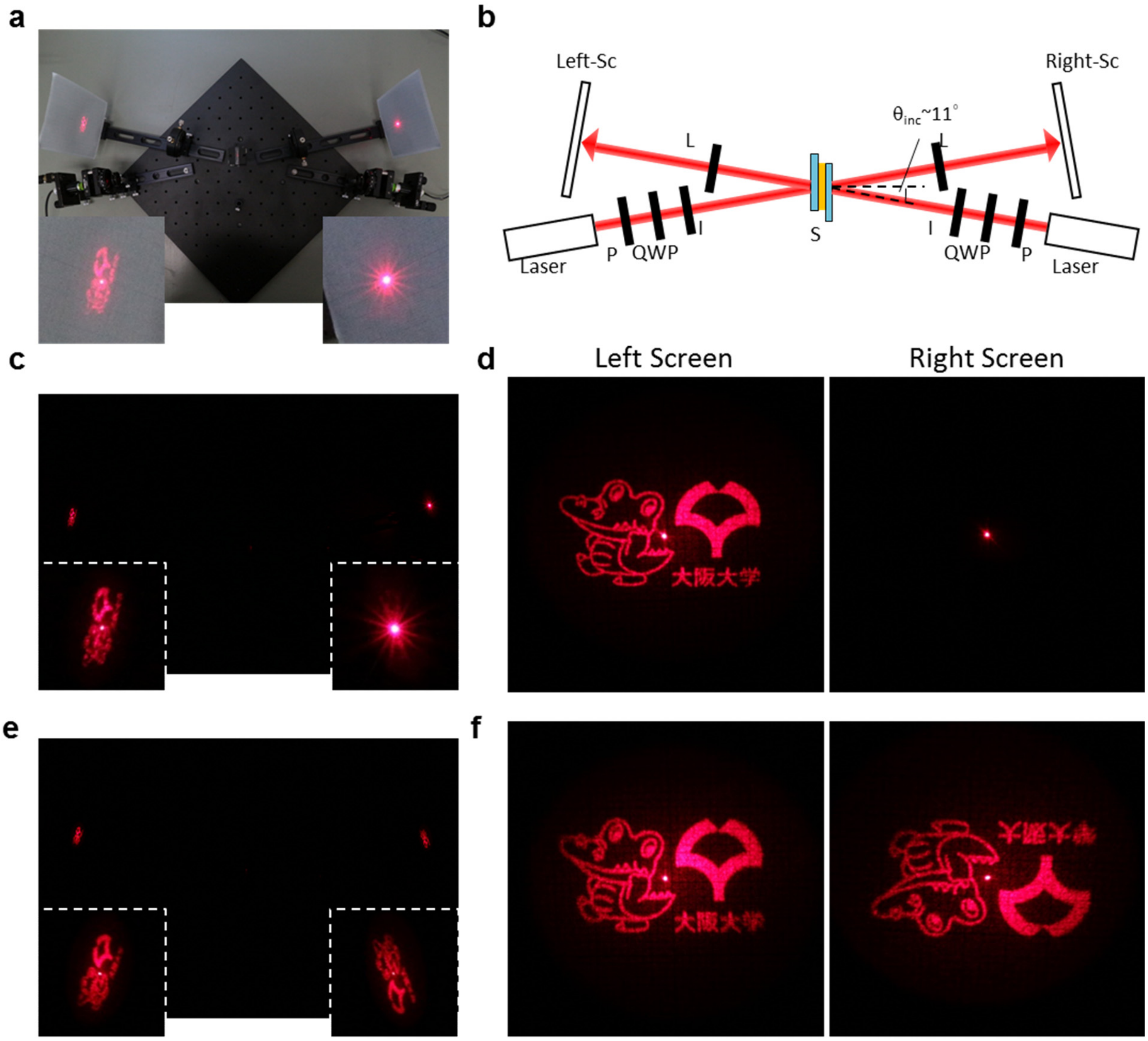

Fig. S7. Effect of symmetric and asymmetric surface alignment on hologram quality. a,b Photograph (a) and schematic image (b) of experimental setup. P: Polarizer; QWP: Quarter wave plate; I: Iris; S: Sample; L : Lens (f = 150 mm); Sc: Screen. Inset: magnified view of screens. **c-f**, Reconstruction of asymmetric (**c,d**) and symmetric cell (**e,f**), and close-up images of reconstructed wavefronts projects on the screens (**d,f**).

7. Design of the pseudo-color, chiral binary hologram

The reflectance spectra of the six-layers were acquired using the same setup as that described in Section 2. As shown in fig. S8, three reflection bands appeared at approximate wavelength ranges of 450–470, 520–550, 620–660 nm. The sample was confirmed to reflect light at 455, 532, and 640 nm, which are the main emission wavelengths of the laser used to illuminate the sample.

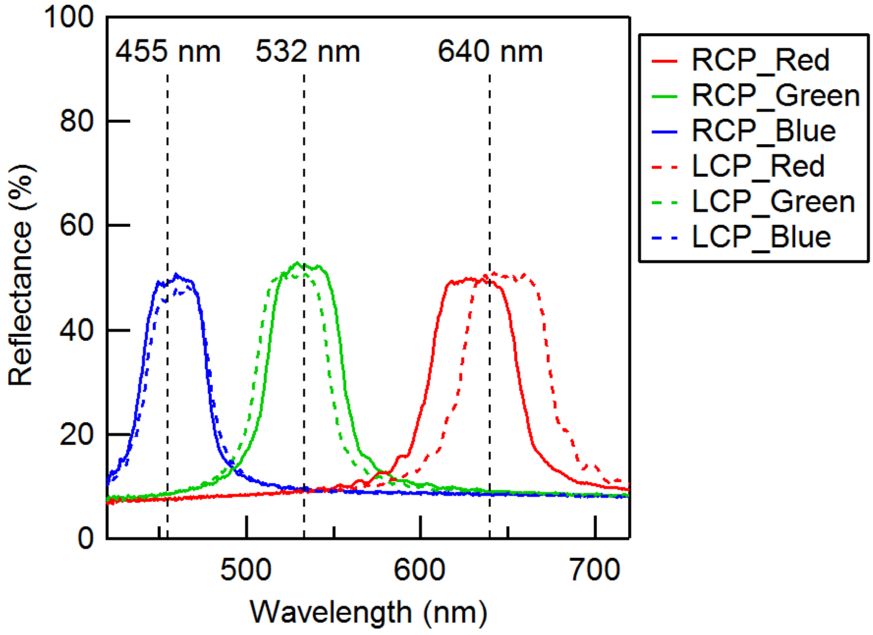

Fig. S8. Reflection spectra of the six-layered ChLC hologram device ..

Fig. S9 shows the target image and phase distributions for right (a-c) and left (d-f) handed ChLCs, designed to operate at three different wavelengths (455, 532, 640 nm). Before calculating the helix phase distribution according to the G-S algorithm, chromatic aberration was corrected by expanding the target images in the Fourier plane by λ_0/λ_1, where λ_1 is the wavelength of the lasers (455, 532, 640 nm) and λ_0 is the reference wavelength (640 nm). Also, the optical phase was obtained by multiplying a factor of ±0.5 to the optical phase, since the relationship between the optical phase and helix phase reverses sign depending on the handedness of the helical structure [15]. Each layer was confirmed to operate as designed, as shown in Fig. 5b of the manuscript.

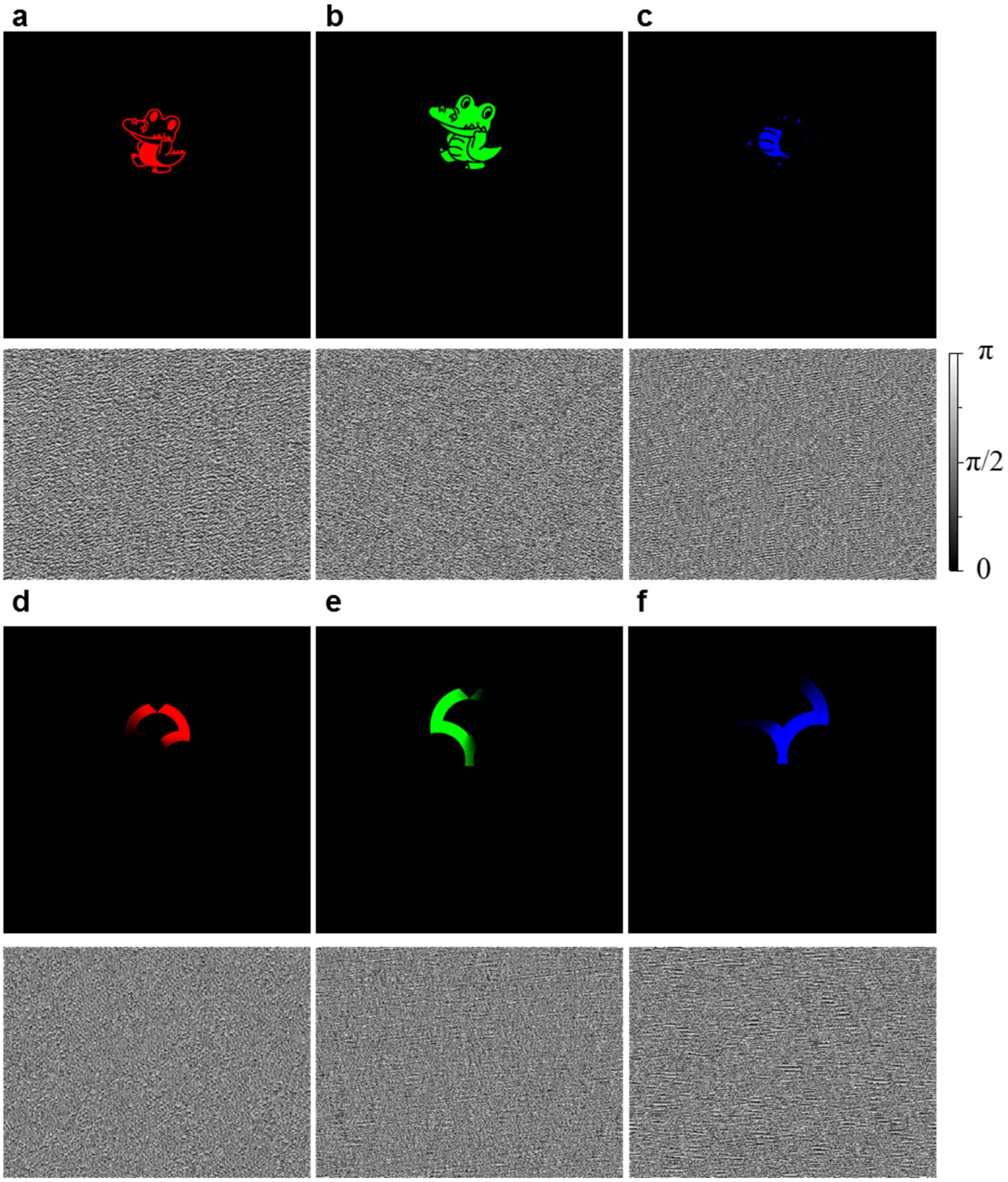

Fig. S9. Hologram design and calculation for color and polarization encoded hologram. a-c, Target image and phase distributions for right-handed ChLCs designed to operate at 455 nm (**a**), 532 nm (**b**), and 640 nm (**c**). **d-f**, S Target image and phase distributions for left-handed ChLCs designed to operate at 455 nm (**d**), 532 nm (**e**), and 640 nm (**f**).

Supplementary Movie 1. Pseudo-color image generation from six-layered hologram device. The six-layered device containing red, green and blue reflecting layers of left and right-handednesses yield different pseudo-color images depending on the incident circular polarization. In the movie, the projected image is seen to change gradually with the turn of a broadband quarter wavelength placed between the laser source and the sample.

References

[1] D. W. Berreman, “Optics in Stratified and Anisotropic Media: 4×4-Matrix Formulation”, *J. Opt. Soc. Am.* **62**, 502-510 (1972).
